# Adverse Outcome Prediction of Iron Deficiency in Patients with Acute Coronary Syndrome

**DOI:** 10.3390/biom8030060

**Published:** 2018-07-20

**Authors:** Tanja Zeller, Christoph Waldeyer, Francisco Ojeda, Renate B. Schnabel, Sarina Schäfer, Alev Altay, Karl J. Lackner, Stefan D. Anker, Dirk Westermann, Stefan Blankenberg, Mahir Karakas

**Affiliations:** 1 Department of General and Interventional Cardiology, University Heart Center Hamburg, 20246 Hamburg, Germany; t.zeller@uke.de (T.Z.); c.waldeyer@uke.de (C.W.); f.ojeda-echevarria@uke.de (F.O.); r.schnabel@uke.de (R.B.S.); sar.schaefer@uke.de (S.S.); alevaltay@web.de (A.A.); d.westermann@uke.de (D.W.); s.blankenberg@uke.de (S.B.); 2 German Center for Cardiovascular Research (DZHK), Partner Site Hamburg, Lübeck, Kiel, 20246 Hamburg, Germany; Karl.Lackner@unimedizin-mainz.de (K.J.L.); s.anker@cachexia.de (S.D.A.); 3 Department of Laboratory Medicine, University Medical Center, Johannes Gutenberg University Mainz, 55131 Mainz, Germany; 4 Department of Cardiology (CVK) and Berlin-Brandenburg Center for Regenerative Therapies (BCRT), 13353 Berlin, Germany

**Keywords:** iron deficiency, acute coronary syndrome, biomarker, prognosis

## Abstract

Acute myocardial infarction remains a leading cause of morbidity and mortality. While iron deficient heart failure patients are at increased risk of future cardiovascular events and see improvement with intravenous supplementation, the clinical relevance of iron deficiency in acute coronary syndrome remains unclear. We aimed to evaluate the prognostic value of iron deficiency in the acute coronary syndrome (ACS). Levels of ferritin, iron, and transferrin were measured at baseline in 836 patients with ACS. A total of 29.1% was categorized as iron deficient. The prevalence of iron deficiency was clearly higher in women (42.8%), and in patients with anemia (42.5%). During a median follow-up of 4.0 years, 111 subjects (13.3%) experienced non-fatal myocardial infarction (MI) and cardiovascular mortality as combined endpoint. Iron deficiency strongly predicted non-fatal MI and cardiovascular mortality with a hazard ratio (HR) of 1.52 (95% confidence interval (CI) 1.03-2.26; *p* = 0.037) adjusted for age, sex, hypertension, smoking status, diabetes, hyperlipidemia, body-mass-index (BMI) This association remained significant (HR 1.73 (95% CI 1.07–2.81; *p* = 0.026)) after an additional adjustment for surrogates of cardiac function and heart failure severity (N-terminal pro B-type natriuretic peptide, NT-proBNP), for the size of myocardial necrosis (troponin), and for anemia (hemoglobin). Survival analyses for cardiovascular mortality and MI provided further evidence for the prognostic relevance of iron deficiency (HR 1.50 (95% CI 1.02–2.20)). Our data showed that iron deficiency is strongly associated with adverse outcome in acute coronary syndrome.

## 1. Introduction

Maintenance of the iron homeostasis is essential for metabolic and physiological processes. Iron plays a critical role in erythropoiesis, is incorporated into erythroblasts and reticulocytes [1], and has a crucial role in oxygen transport and oxygen storage [2]. Moreover, iron is essential for cardiac and skeletal muscle metabolism, the synthesis and degradation of proteins, lipids, ribonucleic acids, and mitochondrial function [3].

Research has now focused on non-traditional cardiovascular risk factors and the roles of iron and iron deficiency (ID) in patients with cardiovascular disease [4,5]. Iron deficiency is a common disorder and is distinguished into absolute and functional ID [6]. Absolute ID reflects depleted iron stores, often with intact iron homoeostasis mechanisms and erythropoiesis, while functional ID, in contrast, reflects inadequate iron supply despite normal or abundant body iron stores [7]. In chronic diseases, absolute ID is typically diagnosed with low cut-off ferritin values (100 µg/L) and is distinguished from functional ID, which is diagnosed with normal serum ferritin (100–300 µg/L) and low transferrin saturation (TSAT) (<20%) [8,9]. This definition of ID is commonly used in heart failure and was successfully validated in clinical trials [10].

While iron deficient heart failure patients are at increased risk of future cardiovascular events and see improvement with intravenous iron supplementation, the clinical relevance of iron deficiency in acute coronary syndrome (ACS) remains unclear [11,12].

In this study, we aimed (i) to assess the prevalence of ID in ACS, (ii) to evaluate the prognostic value of ID in 836 patients with ACS, and (iii) to investigate whether the potential clinical impact is independent of the presence of anemia, size of myocardial infarction (MI), and systolic heart failure.

## 2. Materials and Methods

### 2.1. Study Population

The AtheroGene study involves all subjects with ACS who underwent cardiac catherization at two sites between June 1999 and March 2000 [13,14]. Subjects with relevant valvular heart disease, cardiomyopathy, intake of anticoagulation during the last month, cancer, trauma, and fever were excluded. As a second step, those with missing baseline and follow-up data were excluded, resulting in 1303 patients with ACS. Finally, those with incomplete laboratory information were ruled out. This resulted in a total of 836 patients for the current analysis.

Informed consent was obtained by all patients. AtheroGene is in accordance with the Declaration of Helsinki and obtained approved by the Ethics Board of the Johannes Gutenberg University of Mainz and of the Physicians’ chamber of the State of Rhineland-Palatinate (Germany) under the number 837.057.99.

### 2.2. Data Collection

Baseline information was obtained using standardized questionnaires and hospital charts. Regarding baseline medication, there was no difference between incident cases and non-cases. Coronary artery disease (CAD) was diagnosed when cardiac catherization revealed stenosis >30% in a major coronary artery. Braunwald criteria were used in order to define unstable angina, while diagnosis of acute MI (AMI) was based on cardiovascular guideline definitions [15]. All patients were followed-up for a mean of 4.0 years after discharge. For follow-ups, patients either presented themselves at our clinic (87.2%) or were interviewed by telephone by trained medical staff. Information about the cause of death or clinical events was obtained from hospital or general practitioner charts, using a mailed standardized questionnaire and adjudicated by two independent trained physicians. Deaths and respective causes (international classification of diseases (ICD)-9 codes 390-459: ICD-10 codes I0-I99 and R57.0) were validated using death certificates. Cardiovascular mortality (death certificate) and non-fatal MI were used as combined endpoints.

### 2.3. Laboratory Methods

Baseline biobanking was performed immediately before cardiac catherization. Levels of ferritin, iron, and transferrin were measured in blinded fashion, using automated immunoassays (Roche Cobas Integra 400, Basel, Switzerland). Other laboratory values were measured routinely.

The definition of iron deficiency was based on serum ferritin and TSAT: serum ferritin had to be either <100 μg/L, or 100–299 μg/L with TSAT < 20%. Transferrin saturation was calculated as the ratio of serum iron (μg/dL) and total iron binding capacity (TIBC) (μg/dL) multiplied by 100 and expressed as a percentage. Anemia was defined as hemoglobin level <12.0 g/dL [10].

### 2.4. Statistical Methods

Patient population was described giving baseline characteristics, while the correlation of iron parameters with variables of interest is shown by calculating Spearman correlation coefficients.

Hazard ratios for the outcome measure future cardiovascular mortality/non-fatal MI according presence and absence of ID were estimated by Cox regression models adjusted for potential confounders. Two adjusted models were constructed: (i) model 1 was adjusted for age (years), sex, hypertension, smoking status, diabetes, hyperlipidemia, body-mass-index (BMI); and (ii) model 2 additionally for hemoglobin, log N-terminal pro B-type natriuretic peptide (NT-proBNP), and log (troponin I). Moreover, survival curves according to ID were produced.

Statistics were calculated with R 3.2.4 (http://www.r-project.org/). All *p*-values below 0.05 were determined statistically significant.

## 3. Results

**Figure 1:**
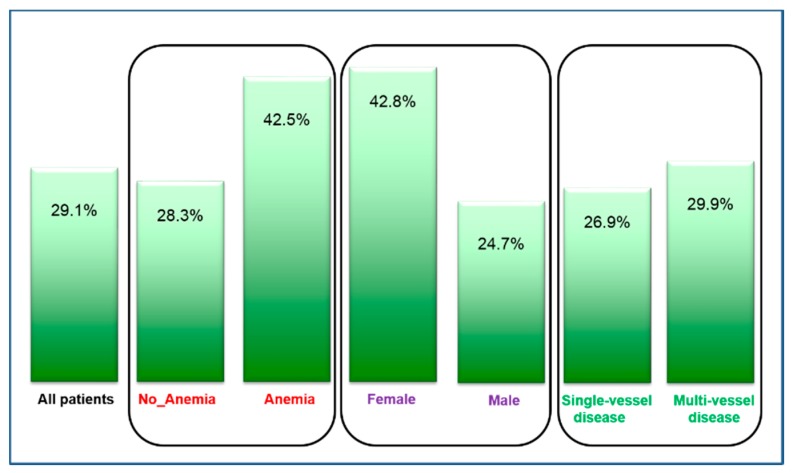
Prevalence of iron deficiency across subgroups of acute coronary syndrome.

Table 1 shows the main sociodemographic and laboratory characteristics at baseline in 836 patients with ACS. The mean age was 63.0 years, and participants were predominantly men (76%). While median ferritin was 236.2 µg/L and median TSAT was 25.5%, the prevalence of iron deficiency at the time of blood draw was 29.1%. Figure 1 shows the prevalence of ID in subgroups across ACS. A total of 29.1% of our sample was categorized as iron deficient. The prevalence of iron deficiency was clearly higher in women (42.8%, *p* < 0.001) and in patients with anemia (42.5%, *p* < 0.001), but only slightly higher in those with multi-vessel CAD compared to one-vessel CAD (29.9% and 26.9%, not significant).

In order to assess the correlation of iron parameters with conventional and emerging cardiovascular risk factors, Spearman rank correlation coefficients (R) were calculated and yielded moderate correlations, if at all (Table 2). Both iron parameters were significantly correlated with male sex, dyslipidemia, troponin I, and haemoglobin, while only TSAT was correlated with NT-proBNP, and only ferritin was correlated with BMI. Nevertheless, all correlations were negligible (below ±0.33). Interestingly, although significantly correlated, the correlation of TSAT and ferritin with hemoglobin was rather weak (TSAT: R = 0.08, *p* = 0.038; ferritin: R = 0.09, *p* = 0.012).

During a median follow-up of 4.0 years, 111 patients (13.3%) experienced cardiovascular death or non-fatal MI. Cox regression analyses were performed to assess whether ID was associated with cardiovascular death and non-fatal MI, (Table 3). Iron deficiency at baseline strongly predicted non-fatal MI and cardiovascular mortality in multivariate Cox regression analyses adjusted for sex, age, and additional cardiovascular risk factors (hazard ratio (HR) 1.52 (95% confidence interval (CI) 1.03–2.26; *p* = 0.037)). Remarkably, this association remained significant (HR 1.73 (95% CI 1.07– 2.81; *p* = 0.026)) after an additional adjustment for surrogates of systolic heart function (NT-proBNP), for the size of myocardial necrosis (troponin I), and for anemia (hemoglobin).

**Figure 2:**
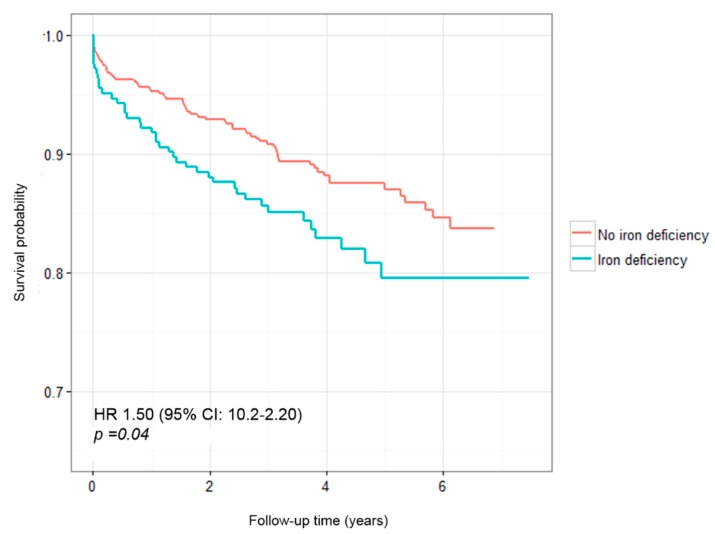
Survival curves for non-fatal myocardial infarction and cardiovascular mortality according to iron status.

The survival curves for cardiovascular mortality and MI according to the presence and absence of ID at baseline (Figure 2) also evidenced the prognostic relevance of ID (HR 1.50 (95% CI 1.02–2.20); *p* = 0.04).

## 4. Discussion

In this study, we evaluated the predictive role of ID for cardiovascular mortality and non-fatal MI in patients with ACS. To our knowledge, this is the first study to evaluate the impact of ferritin and TSAT-based ID in a cohort of documented ACS patients.

We report three major findings: first, ID has a high prevalence in ACS patients. At the time immediately before catherization, when blood was drawn, 29.1% of all subjects were iron deficient. This rate was even higher in women (42.8%) and in patients presenting anemia (42.5%).

Secondly, although the prevalence of iron deficiency was one third higher in the anemic subcohort, TSAT and ferritin showed only a weak correlation with hemoglobin in the Spearman rank analyses.

Thirdly, ID strongly predicted cardiovascular mortality and non-fatal MI in multivariate Cox regression analyses, even after an adjustment was made for surrogates of systolic heart function, size of myocardial necrosis, and anemia. The risk of cardiovascular mortality and non-fatal MI in the 4 years after ACS was 73% higher in those who were iron-deficient at time of ACS (HR 1.73 (95% CI 1.07–2.81; *p* = 0.026)).

### 4.1. Pathophysiological Implications and Clinical Impact

The high prevalence of ID in our population seems in accordance with a previous small cross-sectional study: by obtaining bone marrow aspirates from 65 patients with stable CAD undergoing cardiac surgery, and 10 healthy controls, bone marrow iron depletion was demonstrated in 31 patients with CAD but in none of the controls [16]. Nevertheless, prevalence seems to be slightly lower than in heart failure [17,18]: in a respective study, out of 546 patients with stable systolic heart failure as determined by reduced ejection fraction, 37% of individuals were found to be iron deficient.

The negligible correlation between TSAT and ferritin with hemoglobin clearly supports the hypothesis that ID is a distinct disease, with anemia as a sign of advanced and long-lasting ID. This finding is supported by sub-analyses in clinical trials. In the FAIR-HF trial, two thirds of patients had CAD as an underlying etiology of heart failure [10]. The beneficial effects of intravenous iron supplementation in this trial were independent of hemoglobin levels as treatment with ferric carboxymaltose was beneficial to patients with and without anemia.

Regarding the finding that ID is associated with an adverse outcome in the mid-term, this was also pointed out by a previous report within the LURIC study [19]. Although the study was of cross-sectional design, was not conducted in patients with ACS but stable CAD, and had authors who did not use classical definition of ID, iron depletion was independently associated with angiographic CAD.

There are three main issues that need to be considered in relation to the ACS pathophysiology. First, accumulated experimental data clearly show that during hypoxic conditions, mainly caused by underlying ischaemic disease, iron impairs the viability of cardiomyocytes [20]. Iron seems to act as a protective agent, since the levels of cellular atrophy decreases in hypoxic rat cardiomyocytes treated with iron salt [19]. Several recent studies have established this link between hypoxia, myocardial ischemia, and iron metabolism: Isoda et al. showed that levels of iron-regulating hormone hepcidin, which strongly correlate with ferritin levels, show an abrupt increase of more than 100-fold in human cardiomyocytes within one day after myocardial infarction [21]. This was also shown in a rat model of hypoxia and a rat model of myocardial infarction, resulting in a strong upregulation of hepcidin expression on messenger RNA (mRNA) and protein levels [22,23]. Iron-deficient patients are not able to show such a striking increase in cardiac iron, and thereby might be prone to more substantial atrophy and cardiac apoptosis during AMI.

Second, iron is critically involved in the citric acid cycle, and modulates the expression of the enzyme aconitase by modulating iron regulatory proteins [1,2,24,25]. The supplementation of iron results in an increased mitochondrial adenosine triphosphate (ATP) formation via oxidative phosphorylation, and in reduced glucose utilization. In iron-deficient subjects, adverse glycolysis and lactate formation increases in order to compensate for decreased ATP production, and might enhance ischemic stress and the apoptosis of cardiomyocytes [26]. A most recent study within mice with cardiomyocyte-targeted deletion of iron-regulatory proteins (IRP) showed that the activity of the iron-sulphur cluster-containing complex I of the mitochondrial electron transport chain was reduced in the left ventricles of these mice compared with wild-type [27]. These mice were unable to increase left-ventricular systolic function in response to an acute dobutamine challenge and developed more severe left-ventricular dysfunction with increased mortality after AMI. Interestingly, intravenous injection of iron (ferric carboxymaltose) replenished cardiac iron stores, restored mitochondrial respiratory capacity and inotropic reserve, and attenuated adverse remodeling after AMI in IRP-deleted mice, but not in control mice.

Third, emerging data indicates that macrophages accumulating in the infarcted myocardium take up iron, and in consequence shift their immunological profile towards an anti-inflammatory phenotype [26]. One may hypothesize that iron has protective immunomodulatory effects on macrophages, resulting in an improved infarct healing and beneficial global left-ventricular remodeling in case of ACS [28].

A recent clinical study confirms improved remodeling as a potential mechanism of action of iron in AMI [26]: seventeen study patients (54 ± 9 years, 88% male) underwent cardiac magnetic resonance imaging (MRI) using an iron-rich contrast agent and were matched with 22 historic controls (57 ± 9 years, 77% male), with conventional contrast agent used. All of these patients with primarily reperfused ST-elevation myocardial infarction underwent multi-parametric cardiac MRI studies in the first week and three months after acute MI. There was a more pronounced decrease in infarct size in the iron-treated group (−10.3 ± 5.4% vs., −7.0 ± 8.4%, *p* = 0.050) in addition to a significant decrease in both, endocardial extent, and prevalence of transmural infarctions in iron but not in control patients. A significant decrease in left-ventricular end systolic volume was only seen in the iron-treated group (71 ± 25 mL to 59 ± 25 mL, *p* = 0.002). A significant improvement in ejection fraction was seen in both groups and was more pronounced in the iron-treated group, although not reaching a level of statistical significance. The most important limitation was that the supplementation with iron was irrespective of iron status. One may hypothesize that the beneficial effects of iron in this study would have been much larger, if only subjects with evidence of ID were supplemented.

Our data show that ID is a wide-spread comorbidity in ACS, which is strongly associated with adverse outcome in the mid-term, independent of systolic heart function, the size of myocardial necrosis, and anemia. The potential clinical significance of this project is substantial, regarding the fact that AMI remains a leading cause of morbidity and mortality worldwide. Approximately 450,000 people in the United States die from coronary disease per year, and the 2013 overall rate of death attributable to cardiovascular disease was 222.9 per 100,000 Americans [29]. Based on our results and evidence from the literature, the favorable safety profile of intravenous iron supplementation, and the large evidence of anemia-independent efficacy of iron supplementation in systolic heart failure, we set up the multicentre, placebo-randomized, controlled CAYAN (comprehensive management of iron deficiency in myocardial infarction)-trial [9]. The trial was registered and assigned the European clinical trials database number 2015-005744-34. Within this multicenter trial, patients with clinically relevant AMI and concomitant ID will be enrolled and randomly assigned to receive repletion doses of intravenous iron (ferric carboxymaltose) or saline (placebo). For the primary end point (change in left-ventricular ejection fraction from baseline to month 4), all patients will undergo CMR at the baseline and at the follow-up.

### 4.2. Study Limitations

Our study has limitations that need to be addressed. First, we did not collect information regarding medication during the follow-up. Second, like in most ACS populations, women are clearly underrepresented. Furthermore, Cox regression analyses, in particular model 2, should be interpreted with caution, due to a possible over-adjustment.

## 5. Conclusions

Iron deficiency is a wide-spread comorbidity in ACS. It is strongly associated with adverse outcome in the mid-term, independent of systolic heart function, size of myocardial necrosis, and anemia. This first report on the prospective relevance of ID based on ferritin and TSAT in ACS paved the way to the first placebo-controlled multicenter trial.

## Figures and Tables

**Table 1:** Baseline characteristics of the study patients.

*n*	836
Age (years) *	63 (54, 70)
Male sex (%)	76.0
BMI (kg/m²) *	27.2 (24.9, 29.7)
Smoker (%)	27.5
Diabetes (%)	20.0
Hypertension (%)	68.2
Hyperlipidemia (%)	63.4
History of MI (%)	37.0
Total cholesterol (mg/dL) *	202.0 (176.0, 233.3)
HDL-C (mg/dL) *	46.0 (40.0, 56.0)
LDL-C (mg/dL) *	128.0 (104.0, 155.0)
Troponin I (ng/mL) *	0.1 (0, 2.0)
NT-proBNP (pg/mL) *	429.0 (158.7, 1288.3)
CRP (mg/dL) *^,#^	6.3 (2.5, 20.2)
Ferritin (µg/L) *	236.2 (117.3, 387.2)
TSAT (%) *	25.5 (13.5, 48.0)
Iron deficiency (%)	29.1
Hemoglobin (g/dL) *	14.1 (13.1, 15.0)

BMI = body-mass-index, HDL-C = high-density lipoprotein-cholesterol, LDL-C = low-density lipoprotein-cholesterol, CRP = C-reactive protein, MI = myocardial infarction, TSAT = transferrin saturation, NT-proBNP = N-terminal pro B-type natriuretic peptide. * Median 25th and 75th quartile cut-point; ^#^ only available in a subset of patients.

**Table 2:** Partial Spearman rank correlation coefficients.

	Ferritin (*p*-Value)	TSAT (*p*-Value)
Male gender	0.26 (<0.001)	−0.13 (<0.001)
Age	−0.04 (0.20)	−0.04 (0.27)
Smoking status	−0.02 (0.59)	0.06 (0.12)
Diabetes	0.05 (0.12)	−0.05 (0.16)
Hypertension	−0.01 (0.71)	<0.01 (0.98)
History of MI	−0.05 (0.18)	0.05 (0.16)
Hyperlipidemia	−0.09 (0.007)	0.14 (<0.001)
BMI	0.08 (0.030)	−0.05 (0.18)
Troponin I	0.14 (<0.001)	−0.31 (<0.001)
NT-proBNP	0.05 (0.22)	−0.24 (<0.001)
Hemoglobin	0.09 (0.012)	0.08 (0.038)

**Table 3:** Association of iron deficiency with non-fatal MI and cardiovascular mortality during 4-year follow-up.

	HR	95% CI	*p*-Value
Model 1	1.52	1.03–2.26	0.037
Model 2	1.73	1.07–2.81	0.026

Model 1: adjusted for age, sex, hypertension, smoking status, diabetes, hyperlipidemia, BMI; Model 2: Model 1 additionally adjusted for hemoglobin, log (NT-proBNP), log (troponin I). HR = hazard ratio, CI = confidence interval.
